# Acute large-dose exposure to organophosphates in patients with and without diabetes mellitus: analysis of mortality rate and new-onset diabetes mellitus

**DOI:** 10.1186/1476-069X-13-11

**Published:** 2014-03-06

**Authors:** Shou-Hsuan Liu, Ja-Liang Lin, Hsin-Lan Shen, Chih-Chun Chang, Wen-Hung Huang, Cheng-Hao Weng, Ching-Wei Hsu, I-Kuan Wang, Chih-Chia Liang, Tzung-Hai Yen

**Affiliations:** 1 Department of Nephrology and Division of Clinical Toxicology, Chang Gung Memorial Hospital and Chang Gung University, Taipei, Taiwan; 2 Department of Craniofacial Orthodontics, Chang Gung Memorial Hospital, Linkou, Taiwan; 3 Department of Clinical Pathology, Far Eastern Memorial Hospital, Banciao, New Taipei City, Taiwan; 4 Department of Nephrology, China Medical University Hospital and China Medical University, Taichung, Taiwan; 5 Kidney Research Center, Chang Gung Memorial Hospital, Linkou, Taiwan; 6 Center for Tissue Engineering, Chang Gung Memorial Hospital, Linkou, Taiwan

**Keywords:** Organophosphate poisoning, Suicide, Diabetes mellitus, Mortality, New-onset diabetes mellitus

## Abstract

**Background:**

We investigated the mortality rates of patients with and without diabetes mellitus after acute large-dose exposure to organophosphate insecticides. All patients without diabetes mellitus were traced to examine the long-term risk of new-onset diabetes mellitus. Previous reports indicated that organophosphate exposure might increase the risk of new-onset diabetes mellitus.

**Methods:**

We analyzed the records of 118 patients referred to Chang Gung Memorial Hospital for management of intentional organophosphate poisoning between 2000 and 2011. Patients were stratified by diabetes mellitus status. Demographic, clinical, laboratory and mortality data were analyzed.

**Results:**

Most patients were middle aged (53.45 ± 16.20 years) and male (65.3%) and were referred to our hospital after a relatively short amount of time had elapsed since poisoning (median 3.0 hours). 18 (15.2%) of 118 patients died, including 15 (13.8%) of 109 patients without diabetes mellitus and 3 (33.3%) of 9 with diabetes mellitus. There was no significant difference in mortality between these groups (P = 0.117). In a multivariate Cox regression model, hypotension (P = 0.000), respiratory failure (P = 0.042), coma (P = 0.023), and corrected QT interval prolongation (P = 0.002) were significant risk factors for mortality. Conversely, diabetes mellitus status was not a significant variable in this model. At routine outpatient follow up a median of 1.25 months post exposure, random blood glucose measurements gave no evidence of new-onset diabetes in patients without pre-existing diabetes.

**Conclusions:**

Diabetes mellitus status might not increase mortality risk following acute large-dose exposure to organophosphates, and the risk of new-onset diabetes mellitus also might be minimal in the short term. Larger prospective studies with formal testing for diabetes at later times post-exposure are required.

## Background

Intentional ingestion of organophosphate insecticide causes many deaths each year in Taiwan [1]. Chronologically, the 3 different clinical syndromes after acute organophosphate intoxication include (1) early acute cholinergic crisis due to acetylcholinesterase suppression, (2) intermediate syndrome (0.5–7 days) that has an unclear underlying mechanism, and (3) delayed polyneuropathy (6–21 days) explained by the inhibition of neuropathy target esterase. Acute cholinergic crisis [2] includes signs and symptoms resulting from hyperstimulation of muscarinic receptors in the parasympathetic system (e.g., bradycardia, bronchospasm, bronchorrhea, hypotension, diarrhea, vomiting, miosis, lachrymation, salivation, and urination), nicotinic receptors in the sympathetic system (e.g., hypertension, tachycardia, mydriasis, and sweating), nicotinic receptors at the neuromuscular junction (e.g., muscle weakness, paralysis, and fasciculations), and both central muscarinic and nicotinic receptors in the central nervous system (e.g., confusion, agitation, coma, and respiratory failure).

We performed this study because, as demonstrated previously, diabetes mellitus could modify drug pharmacokinetics by altering hepatic drug-metabolizing enzyme activity [3,4]. In addition, Starr et al [5] found that increased blood-brain barrier permeability with magnetic resonance imaging was detected in patients with type II diabetes or white matter hyperintensities. Increased permeability of the blood-brain barrier might account for some of the cerebral effects of type II diabetes, and so possibly also for the effect of other conditions that affect the microvasculature (like hypertension), on the brain. Therefore, we hypothesized that diabetic patient with organophosphates poisoning might suffer from a higher mortality rate than patients without diabetes.

We also performed this study because several reports have shown that organophosphates might have a special effect on the mammalian pancreas [6], especially on glucose homeostasis and the possibility of new-onset diabetes mellitus [7,8]. Therefore, we investigated the mortality rates of patients with and without diabetes mellitus after acute large-dose exposure to organophosphate insecticides. In addition, all patients without diabetes mellitus were traced to examine the short to medium term risk of new-onset diabetes mellitus.

## Methods

This retrospective observational study complied with the Declaration of Helsinki guidelines and was approved by the Medical Ethics Committee of Chang Gung Memorial Hospital, a tertiary referral center located in the northern part of Taiwan. Because this study was a retrospective review of existing data, Institutional Review Board approval was obtained, but without specific informed consent from the patients. However, informed consent of acute organophosphate poisoning risk and all treatment modalities (including cardiopulmonary cerebral resuscitation, etc.) was obtained from all patients at their initial admission. In addition, all individual information was securely protected (by delinking identifying information from the main data set) and was available to investigators only. Furthermore, all data were analyzed anonymously. The Institutional Review Board of Chang Gung Memorial Hospital specifically waived the need for consent. Finally, all primary data were collected according to the Strengthening the Reporting of Observational Studies in Epidemiology guidelines. This policy was based on previous publications [9,10].

### Patients

We analyzed the records of 118 patients with intentional organophosphate poisoning examined at Chang Gung Memorial Hospital between 2000 and 2011. The diagnosis of organophosphate poisoning was based on history of exposure, clinical effects, and serum cholinesterase activity. Serum cholinesterase activity was determined using an enzymatic method (normal values: 7–19 U/mL) immediately after the patient arrived at the hospital. The pesticide ingested was determined by history, container label, or product information provided by the patient. A complete clinical profile of each patient was recorded using a standardized form. We obtained the following data for each patient: age, sex, blood pressure, heart rate, type and toxicity classification of the organophosphate, underlying diseases, smoking habits, alcohol consumption, medications, time elapsed between poisoning and hospital arrival, duration of follow-up, clinical manifestations, electrocardiogram results, serum cholinesterase level, hemogram, biochemistry, the detoxification protocol used, and mortality.

### Inclusion and exclusion criteria

All patients aged >18 years diagnosed with organophosphate poisoning at Chang Gung Memorial Hospital between 2000 and 2011 were eligible for inclusion in this study. Patients were excluded if they were aged <18 years or did not have suppressed serum cholinesterase levels despite suspicions of exposure.

### Detoxification protocols

The protocols [10] used to treat patients included gastric lavage with large amounts of normal saline, followed by infusion of 1 g/kg activated charcoal and 250 mL of magnesium citrate administered via a nasogastric tube. Magnesium citrate was used to prevent constipation after charcoal administration. Patients were also treated with specific antidotes including atropine and oximes. Intravenous atropine was administered at a starting dose of 2 mg every 1–2 hours, and dosing was titrated to the clearing of respiratory secretions and cessation of bronchoconstriction. All patients with evidence of cholinergic toxicity were given pralidoxime therapy (1 g every 4 hours administered intravenously).

### Definition of clinical events

We classified organophosphate toxicity using the following formal World Health Organization recommendations: class Ia (extremely hazardous), class Ib (highly hazardous), class II (moderately hazardous), class III (slightly hazardous), and class U (unlikely to present acute hazard) [11]. Acute renal failure was diagnosed if the serum creatinine level increased to >1.27 mg/dL in men or 1.03 mg/dL in women. Acute respiratory failure was defined as a condition of respiratory insufficiency requiring intubation and mechanical ventilation for more than 24 hours, regardless of the fraction of inspired oxygen [12]. Hypotension was defined as systolic blood pressure <90 mm Hg. Structural heart disease was defined as noncoronary cardiovascular disease processes and related interventions [13]. Use of medications that might be associated with corrected QT interval (QTc) prolongation was recorded. In this regard, the antiarrhythmic agents included class Ia, class Ic, and class III agents [14]. Antimicrobials included macrolides/ketolides, certain fluoroquinolones, certain antimalarials, pentamidine, and azole antifungals [15]. Antipsychotics and antidepressants included (1) typical antipsychotics such as chlorpromazine, pimozide, thioridazine, perphenazine, trifluoperazine, haloperidol, and droperidol; (2) atypical antipsychotics such as clozapine, quetiapine, risperidone, sultopride, ziprasidone, and loxapine; (3) tricyclic antidepressants such as amitriptyline, amoxapine, clomipramine, desipramine, citalopram, doxepin, imipramine, nortriptyline, and trimipramine; and (4) other antidepressants such as fluoxetine, sertraline, and venlafaxine [16]. New-onset diabetes mellitus was defined as casual plasma glucose ≥200 mg/dL [17,18] that occurred in patients without pre-existing diabetes mellitus.

### Statistical analysis

Continuous variables are expressed as the mean and standard deviation and categorical variables as the number with percentage in brackets. All data were tested for normality of distribution and equality of standard deviations before analysis. For comparisons between patient groups, we used Student's *t*-test for quantitative variables and chi-square or Fisher's exact test for categorical variables. Mortality data were compared using the Kaplan-Meier method and significance was tested using a log rank test. An initial univariate Cox regression analysis was performed to compare the frequency of possible risk factors associated with mortality. To control for possible confounding factors, a multivariate Cox regression analysis (backward stepwise approach) was performed to analyze factors that were significant in univariate models (P < 0.05) and met the assumptions of a proportional hazards model. Results that rejected the null hypothesis with 95% confidence were considered significant. All analyses were performed using SPSS, version 12.0 for Windows (SPSS Inc., Chicago, Illinois, USA).

## Results

Table 1 shows the baseline characteristics of the 118 patients with organophosphate poisoning overall and by diabetes mellitus status. Of the 118 patients, 109 were in the non–diabetes mellitus group and 9 were in the diabetes mellitus group. Most of the patients were middle aged (53.45 ± 16.20 years) and men (65.3%) and were referred to our hospital after a relatively short amount of time had elapsed since poisoning (median 3.0 hours). Notably, nearly half of the patients had a history of a mental disorder (43.2%) and were undergoing long-term treatment with antipsychotic and antidepressant medications (25.4%). After analysis, we found that patients with diabetes mellitus were older than patients without diabetes mellitus (52.32 ± 16.04 versus 67.11 ± 11.84 years; P = 0.008). Otherwise, there were no significant differences in baseline variables between the groups (P > 0.05).

**Table 1:** Baseline characteristics of patients with organophosphate poisoning, stratified according to diabetes mellitus status (N = 118)

**Variable**	**Total (N = 118)**	**Non-diabetes mellitus (N = 109)**	**Diabetes mellitus (N = 9)**	**P**
Age, years (mean ± SD)	53.45 ± 16.20	52.32 ± 16.04	67.11 ± 11.84	0.008**
Male, n (%)	77 (65.3)	73 (67.0)	4 (44.4)	0.173
Organophosphate type, n (%):				0.082
Chlorpyrifos	48 (40.7)	44 (40.4)	4 (44.4)	
Profenofos	16 (13.6)	16 (14.7)	0 (0.0)	
Methamidophos	14 (11.9)	14 (12.8)	0 (0.0)	
Mevinphos	10 (8.5)	9 (8.3)	1 (11.1)	
Dimethoate	9 (7.6)	8 (7.3)	1 (11.1)	
Malathion	6 (5.1)	5 (4.6)	1 (11.1)	
Parathion	4 (3.4)	4 (3.7)	0 (0.0)	
Acephate	3 (2.5)	2 (1.8)	1 (11.1)	
Trichlorfon	3 (2.5)	3 (2.8)	0 (0.0)	
Terbufos	2 (1.7)	2 (1.8)	0 (0.0)	
Dichlorvos	1 (0.8)	1 (0.9)	0 (0.0)	
Fenamiphos	1 (0.8)	1 (0.9)	0 (0.0)	
Phorate	1 (0.8)	0 (0.0)	1 (11.1)	
Organophosphate toxicity, n (%):				0.206
Ia, extremely hazardous	17 (14.4)	15 (13.8)	2 (22.2)	
Ib, highly hazardous	16 (13.6)	16 (14.7)	0 (0.0)	
II, moderately hazardous	76 (64.4)	71 (65.1)	5 (55.6)	
III, slightly hazardous	9 (7.6)	7 (6.4)	2 (22.2)	
U, unlikely to present acute hazard	0 (0)	0 (0)	0 (0)	
Hypertension, n (%)	21 (17.8)	18 (16.5)	3 (33.3)	0.199
Old stroke, n (%)	10 (8.5)	8 (7.3)	2 (22.2)	0.123
Coronary artery disease, n (%)	3 (2.5)	3 (2.8)	0 (0.0)	0.614
Structural heart disease, n (%)	11 (9.3)	10 (9.2)	1 (11.1)	0.848
Chronic obstructive pulmonary disease, n (%)	9 (7.6)	8 (7.3)	1 (11.1)	0.682
Malignancy, n (%)	3 (2.5)	3 (2.8)	0 (0.0)	0.614
Mental disorder, n (%)	51 (43.2)	46 (42.2)	5 (55.6)	0.437
Smoking habit, n (%)	52 (44.1)	50 (45.9)	2 (22.2)	0.170
Alcohol consumption, n (%)	49 (41.5)	47 (43.1)	2 (22.2)	0.221
Use of medications that might be associated with QTc 7prolongation, n (%):				
Antiarrhythmic agents	20 (16.9)	17 (15.6)	3 (33.3)	0.173
Antipsychotics and antidepressants	30 (25.4)	27 (24.8)	3 (33.3)	0.571
Antimicrobials	10 (8.5)	9 (8.3)	1 (11.1)	0.768

QTc, corrected QT interval.

Note: **P < 0.01.

After ingestion, the patients developed acute cholinergic crisis (Table 2) that included respiratory failure (51.7%), emesis (35.6%), diarrhea (29.7%), hypotension (22.9%), coma (19.5%), acute renal failure (9.3%), and seizure (8.5%), among other conditions. Intermediate syndrome and delayed polyneuropathy were also present in 8.5% and 2.5% of patients, respectively. Nevertheless, there were no significant differences in clinical variables between the groups (P > 0.05). Laboratory analysis revealed suppressed initial and nadir serum cholinesterase levels (3.29 ± 3.53 U/mL and 2.79 ± 3.06 U/mL, respectively) (Table 3). There were no significant differences in laboratory variables between the groups (P > 0.05).

**Table 2:** Clinical manifestations of patients with organophosphate poisoning, stratified according to diabetes mellitus status (N = 118)

**Variable**	**Total (N = 118)**	**Non–diabetes mellitus (N = 109)**	**Diabetes mellitus (N = 9)**	**P**
1. Cholinergic crisis				
Cardiovascular system:				
Systolic BP, mm Hg (mean ± SD)	116.81 ± 23.21	117.46 ± 22.98	108.89 ± 26.00	0.289
Diastolic BP, mm Hg (mean ± SD)	79.68 ± 18.24	80.30 ± 17.88	72.11 ± 21.92	0.197
Heart rate, beats/minute (mean ± SD)	102.04 ± 26.72	103.26 ± 27.75	91.43 ± 11.10	0.270
Hypotension, n (%)	27 (22.9)	24 (22.0)	3 (33.3)	0.437
Gastrointestinal system, n (%):				
Diarrhea	35 (29.7)	35 (32.1)	0 (0.0)	0.053
Emesis	42 (35.6)	40 (36.7)	2 (22.2)	0.383
Respiratory system, n (%):				
Shortness of breath	81 (68.6)	75 (68.8)	6 (66.7)	0.894
Bronchorrhea	72 (61.0)	66 (60.6)	6 (66.7)	0.718
Bronchospasm	67 (56.8)	61 (56.0)	6 (66.7)	0.533
Respiratory failure	61 (51.7)	55 (50.5)	6 (66.7)	0.350
Genitourinary system n (%):				
Acute renal failure	11 (9.3)	10 (9.2)	1 (11.1)	0.848
Central nervous system n (%):				
Seizure	10 (8.5)	8 (7.3)	2 (22.2)	0.123
Coma	23 (19.5)	21 (19.3)	2 (22.2)	0.830
2. Intermediate syndrome, n (%)	10 (8.5)	10 (9.2)	0 (0.0)	0.342
3. Delayed polyneuropathy, n (%)	3 (2.5)	3 (2.8)	0 (0.0)	0.614

SD, standard deviation; BP, blood pressure.

**Table 3:** Laboratory findings of patients with organophosphate poisoning, stratified according to diabetes mellitus status (N = 118)

**Variable (mean ± SD)**	**Total (N = 118)**	**Non–diabetes mellitus (N = 109)**	**Diabetes mellitus (N = 9)**	**P**
Hemoglobin, g/dL	14.33 ± 1.91	14.37 ± 1.95	13.81 ± 1.30	0.428
Blood urea nitrogen, mg/dL	16.74 ± 16.45	16.34 ± 16.71	25.20 ± 4.53	0.463
Creatinine, mg/dL	1.06 ± 0.72	1.05 ± 0.74	1.14 ± 0.47	0.741
Sodium, mEq/L	140.97 ± 3.68	141.04 ± 3.72	140.13 ± 3.40	0.502
Potassium, mEq/L	3.50 ± 0.54	3.48 ± 0.51	3.75 ± 0.76	0.167
Cholinesterase (initial), U/mL	3.29 ± 3.53	3.18 ± 3.24	4.67 ± 6.31	0.254
Cholinesterase (lowest), U/mL	2.79 ± 3.06	2.65 ± 2.65	4.65 ± 6.28	0.400
C-reactive protein, mg/dL	8.18 ± 18.67	8.13 ± 19.31	8.77 ± 15.65	0.616
Amylase, U/L	195.98 ± 167.47	197.62 ± 162.72	176.18 ± 187.21	0.910
Lipase, U/L	113.50 ± 115.10	113.89 ± 117.96	108.74 ± 84.24	0.365
CK-MB, ng/mL	17.98 ± 61.03	17.56 ± 64.77	23.07 ± 48.23	0.691
Troponin-I, ng/mL	0.07 ± 0.13	0.08 ± 0.14	0.05 ± 0.12	0.618
QT interval, ms	369.07 ± 57.59	366.61 ± 58.56	390.57 ± 46.20	0.301
QTc interval, ms	443.18 ± 52.71	440.21 ± 52.96	479.29 ± 53.52	0.236

SD, standard deviation; CK-MB, creatinine kinase MB; QTc, corrected QT interval.

As shown in Table 4, most patients were aggressively treated with gastric lavage with large amounts of normal saline, followed by an infusion of 1 g/kg activated charcoal and 250 mL of magnesium citrate. In addition, nearly all patients were administered systemic atropine and pralidoxime injections. There was no difference in treatment modality between the groups except a slightly lower use of atropine in the diabetes mellitus group compared with the non-diabetes mellitus group (88.9% versus 99.1%; P = 0.023).

**Table 4:** Detoxification protocol and treatment outcome for patients with organophosphate poisoning, stratified according to diabetes mellitus status (N = 118)

**Variable**	**Total (N = 118)**	**Non–diabetes mellitus (N = 109)**	**Diabetes mellitus (N = 9)**	**P**
Gastric lavage, n (%)	95 (80.5)	89 (81.7)	6 (66.7)	0.275
Active charcoal and magnesium citrate, n (%)	88 (74.6)	83 (76.1)	5 (55.6)	0.173
Atropine, n (%)	116 (98.3)	108 (99.1)	8 (88.9)	0.023*
Pralidoxime, n (%)	115 (97.5)	107 (98.2)	8 (88.9)	0.089
Time from poisoning to hospital, hours (median, IQR)	3.0 (2.0, 5.0)	3.0 (2.0, 5.0)	3.5 (2.0, 9.0)	0.893
Duration of follow-up, months (median, IQR)	1.25 (0.40, 9.06)	1.23 (0.43, 8.63)	2.27 (0.25, 15.67)	0.219
Mortality, n (%)	18 (15.3)	15 (13.8)	3 (33.3)	0.117
Cause of death, n (%)				0.333
Cardiogenic	10 (8.5)	8 (7.3)	2 (22.2)	
Respiratory failure	6 (5.1)	5 (4.6)	1 (11.1)	
Sepsis	2 (1.7)	2 (1.8)	0 (0.0)	

IQR, interquartile range.

Note: *P < 0.05.

The median follow-up duration was 1.25 months. 18 (15.3) of 118 patients died, including 15 (13.8%) of 109 patients without diabetes mellitus and 3 (33.3%) of 9 patients with diabetes mellitus. There was no significant difference in mortality between the groups (P = 0.117) (Table 4). Similarly, there was no difference in the cause of mortality between patients with and without diabetes mellitus (P = 0.333).

A multivariate Cox regression model demonstrated that hypotension, respiratory failure, coma, and QTc prolongation were significant risk factors for mortality (Table 5). Notably, diabetes mellitus status was not a significant variable according to this model (P = 0.117).

**Table 5:** Univariate and multivariate Cox regression analysis for mortality (N = 118)

**Variable**	**Univariate analysis OR (95% CI)**	**P**	**Multivariate analysis OR (95% CI)**	**P**
Hypotension	21.596 (6.237–74.780)	0.000***	10.930 (2.961–40.345)	0.000***
Respiratory failure	8.504 (1.955–36.997)	0.004**	4.867 (1.062–22.301)	0.042*
Coma	10.317 (3.857–27.595)	0.000***	3.482 (1.184–10.238)	0.023*
QTc prolongation	9.974 (2.885–34.478)	0.000***	7.459 (2.053–27.099)	0.002**
Diabetes mellitus	2.634 (0.762–9.103)	0.126		

QTc, corrected QT interval.

Note: *P < 0.05, **P < 0.01, ***P < 0.001.

Regarding the possibility of new-onset diabetes mellitus, all 94 of 109 non–diabetes mellitus patients were successfully traced at least 1 time between 2000 and 2012 except 15 patients died during admission. The random blood sugar level was checked during follow-up. At routine outpatient follow up a median of 1.25 months post exposure, random blood glucose measurements gave no evidence of new-onset diabetes in patients without pre-existing diabetes. The other 6 of 9 diabetes mellitus patients were successfully traced as well except 3 patients died during admission (Table 6).

**Table 6:** Random blood glucose test after acute large-dose exposure to organophosphates in patients with and without diabetes mellitus (N = 118)

**Variable (mean ± SD)**	**Total (N = 118)**	**Non–diabetes mellitus (N = 109)**	**Diabetes mellitus (N = 9)**	**P**
Sugar, mg/dL (during hospitalization)	164.3 ± 83.1	144.7 ± 43.6	279.8 ± 10151.0	0.000***
Sugar, mg/dL (after discharge)	142.2 ± 53.7	124.9 ± 22.5	244.0 ± 71.0	0.000***

SD, standard deviation.

Note: ***P < 0.001.

## Discussion

This study is of particular interest because it showed that diabetes mellitus status might not increase mortality risk following acute large-dose exposure to organophosphates (P = 0.117) (Table 4). Furthermore, the risk of new-onset diabetes mellitus after acute intoxication within the short term also might be minimal.

The relationship between organophosphate exposure and subsequent new-onset diabetes mellitus remains unclear. A previous animal study [19] found that dimethoate at subchronic oral doses had the propensity to impair glucose homeostasis and induce significant pancreatic damage, and also provide an account of the associated oxidative damage to pancreatic tissue in adult rats. Additionally [20], the toxicity of diazinon and its metabolites increases in diabetic rats. Slotkin [21] also reported that organophosphate exposure in neonatal rats during a critical developmental window altered the trajectory of hepatic adenylyl cyclase/cyclic AMP signaling, culminating in hyperresponsiveness to gluconeogenic stimuli. Consequently, the rats developed a metabolic dysfunction resembling prediabetes. Finally, it has been shown [22] that low-dose administration of chlorpyrifos in rats resulted in decreased activity of paraoxonase in serum, which was accompanied by an increased level of lipid peroxides. This oxidative stress might explain the further development of arteriosclerosis, hypercholesterolemia, and diabetes mellitus in people exposed to organophosphate compounds.

Shobha et al [23] reported transient glycosuria in 69% of patients after organophosphate poisoning. However, none of the patients had new-onset diabetes mellitus and the pre–hospital discharge blood sugars were in the normal range. Conversely [24-26], organophosphate intoxication presenting as diabetic ketoacidosis has been repeatedly reported in the pediatric population. Using data from the Agricultural Health Study, Saldana et al [7] found that activities involving exposure to agricultural pesticides during the first trimester of pregnancy might increase the risk of gestational diabetes mellitus. In another study using Agricultural Health Study data [8], it was proven that long-term exposure to dichlorvos and trichlorfon among licensed pesticide applicators could increase the risk of incident diabetes mellitus. Finally, a systematic review in 2010 [27] revealed that organophosphates impair the enzymatic pathways involved in the metabolism of carbohydrates within cytoplasm, mitochondria, and peroxisomes. Organophosphates exert this effect by inhibiting acetyl cholinesterase or affecting target organs (such as the pancreas, liver, muscles, and brain) directly. Furthermore, organophosphates also induce cellular oxidative stress by affecting mitochondrial function and therefore disrupt the neuronal and hormonal status of the body. Taken together, both central (pancreas) and peripheral glucose regulatory mechanisms could be impaired after organophosphate exposure [27].

In this study, it was found that the diabetic cohort had lesser load of poisoining as evidenced by the levels of initial cholinesterase (4.67 U/mL versus 3.18 U/mL; P = 0.254) and needed less atropine (99.1% versus 88.9%; P = 0.023). It is also possible that the mortality rates were affected by the less severe load in patient with diabetes mellitus. Nevertheless, the incidence of significant risk factors for mortality such as hypotension (22.0% versus 33.3%), respiratory failure (50.5% versus 66.7%), and coma (19.3% versus 22.2%) were higher in patient with diabetes mellitus. Small patient population may be the reason of non-significance in mortality rate between patient with and without diabetes mellitus.

A multivariate Cox regression analysis (Table 5) confirmed that hypotension, respiratory failure, coma, and QTc prolongation were significant risk factors for mortality after organophosphate poisoning. The associations between both hypotension [28] and coma with mortality were not surprising because both are important vital signs, irrespective of the cause of disease. Similarly, it was not surprising to find an association between respiratory failure and mortality because respiratory failure is a prominent feature of acute organophosphate poisoning with an early central apnea followed by later pulmonary effects [29]. Animal studies have also suggested that organophosphate-induced respiratory failure results from local effects of organophosphates acting on brainstem circuits underlying respiratory rhythmogenesis, and on lung tissues underlying pulmonary secretory, airway, and vascular function [29]. Finally, Chuang et al [30] confirmed that patients with QTc prolongation had a higher mortality rate and a higher incidence of respiratory failure than patients without QTc prolongation.

The retrospective nature, small patient population, short follow-up duration, and inadequate definition of new-onset diabetes mellitus limit the certainty of our conclusions. Therefore, a prospective study with larger patient numbers, longer follow up, and strict assessment of new-onset diabetes mellitus (such as glycated hemoglobin or oral glucose tolerance test) are required to confirm our conclusion.

## Conclusions

Diabetes mellitus status might not increase mortality risk following acute large-dose exposure to organophosphates, and the risk of new-onset diabetes mellitus also might be minimal in the short term. Larger prospective studies with formal testing for diabetes at later times post-exposure are required.

## Abbreviations

QTc: Corrected QT interval.

## Competing interests

The authors have no competing interest to declare.

## Authors’ contributions

All authors have made substantial contributions to conception and design, or acquisition of data, or analysis and interpretation of data. SHL and THY have been involved in drafting the manuscript or revising it critically for important intellectual content. THY have given final approval of the version to be published. All authors agree to be accountable for all aspects of the work in ensuring that questions related to the accuracy or integrity of any part of the work are appropriately investigated and resolved. All authors read and approved the final manuscript.

## References

[B1] Chang S-S Lu T-H Sterne JAC Eddleston M Lin J-J Gunnell D The impact of pesticide suicide on the geographic distribution of suicide in Taiwan: a spatial analysis BMC Publ Health 2012 12 1 260 10.1186/1471-2458-12-260 PMC3351735 22471759

[B2] Eddleston M Buckley NA Eyer P Dawson AH Management of acute organophosphorus pesticide poisoning Lancet 2008 371 597 607 10.1016/S0140-6736(07)61202-1 17706760 PMC2493390

[B3] Dixon RL Hart LG Fouts JR The metabolism of drugs by liver microsomes from alloxan-diabetic rats J Pharmacol Exp Ther 1961 133 7 11 13723159

[B4] Thummel KE Schenkman JB Effects of testosterone and growth hormone treatment on hepatic microsomal P450 expression in the diabetic rat Mol Pharmacol 1990 37 1 119 129 2105452

[B5] Starr JM Wardlaw J Ferguson K MacLullich A Deary IJ Marshall I Increased blood-brain barrier permeability in type II diabetes demonstrated by gadolinium magnetic resonance imaging J Neurol Neurosurg Psychiatry 2003 74 1 70 76 10.1136/jnnp.74.1.70 12486269 PMC1738177

[B6] Lukaszewicz-Hussain A [The effect of organophosphate pesticides on pancreas] Med Pr 2011 62 5 543 550 22312968

[B7] Saldana TM Basso O Hoppin JA Baird DD Knott C Blair A Alavanja MC Sandler DP Pesticide exposure and self-reported gestational diabetes mellitus in the agricultural health study Diabetes Care 2007 30 3 529 534 10.2337/dc06-1832 17327316 PMC3902103

[B8] Montgomery MP Kamel F Saldana TM Alavanja MCR Sandler DP Incident diabetes and pesticide exposure among licensed pesticide applicators: agricultural health study, 1993-2003 Am J Epidemiol 2008 167 10 1235 1246 10.1093/aje/kwn028 18343878 PMC2832308

[B9] Yang CJ Lin JL Lin-Tan DT Weng CH Hsu CW Lee SY Lee SH Chang CM Lin WR Yen TH Spectrum of toxic hepatitis following intentional paraquat ingestion: analysis of 187 cases Liver Int 2012 32 9 1400 1406 10.1111/j.1478-3231.2012.02829.x 22672665

[B10] Liu SH Lin JL Weng CH Yang HY Hsu CW Chen KH Huang WH Yen TH Heart rate-corrected QT interval helps predict mortality after intentional organophosphate poisoning PLoS One 2012 7 5 e36576 10.1371/journal.pone.0036576 22574184 PMC3344908

[B11] World Health Organization http://www.who.int/ipcs/publications/pesticides_hazard_2009.pdf (Accessed on 27 January 2014)

[B12] Luhr OR Antonsen K Karlsson M Aardal S Thorsteinsson A Frostell CG Bonde J Incidence and mortality after acute respiratory failure and acute respiratory distress syndrome in Sweden, Denmark, and Iceland. The ARF Study Group Am J Respir Crit Care Med 1999 159 6 1849 1861 10.1164/ajrccm.159.6.9808136 10351930

[B13] Steinberg DH Staubach S Franke J Sievert H Defining structural heart disease in the adult patient: current scope, inherent challenges and future directions Eur Heart J Suppl 2010 12 Suppl E E2 E9 10.1093/eurheartj/suq012

[B14] Shantsila E Watson T Lip GY Drug-induced QT-interval prolongation and proarrhythmic risk in the treatment of atrial arrhythmias Europace 2007 9 Suppl 4 iv37 iv44 17766322 10.1093/europace/eum169

[B15] Owens RC Jr Nolin TD Antimicrobial-associated QT interval prolongation: pointes of interest Clin Infect Dis 2006 43 12 1603 1611 10.1086/508873 17109296

[B16] Sicouri S Antzelevitch C Sudden cardiac death secondary to antidepressant and antipsychotic drugs Expert Opin Drug Saf 2008 7 2 181 194 10.1517/14740338.7.2.181 18324881 PMC2365731

[B17] Sacks DB Arnold M Bakris GL Bruns DE Horvath AR Kirkman MS Lernmark A Metzger BE Nathan DM Guidelines and recommendations for laboratory analysis in the diagnosis and management of diabetes mellitus Diabetes Care 2011 34 6 e61 e99 10.2337/dc11-9998 21617108 PMC3114322

[B18] Weng CH Hu CC Yu CC Lin JL Yang CW Hung CC Hsu CW Yen TH Immunoglobulin G levels can predict non-diabetic renal disease in patients with type 2 diabetes mellitus J Diabetes 2012 4 1 37 40 10.1111/j.1753-0407.2011.00175.x 22145829

[B19] Kamath V Rajini PS Altered glucose homeostasis and oxidative impairment in pancreas of rats subjected to dimethoate intoxication Toxicology 2007 231 2–3 137 146 17197067 10.1016/j.tox.2006.11.072

[B20] Ueyama J Wang D Kondo T Saito I Takagi K Takagi K Kamijima M Nakajima T Miyamoto K Wakusawa S Hasegawa T Toxicity of diazinon and its metabolites increases in diabetic rats Toxicol Lett 2007 170 3 229 237 10.1016/j.toxlet.2007.03.010 17442507

[B21] Slotkin TA Does early-life exposure to organophosphate insecticides lead to prediabetes and obesity? Reprod Toxicol 2011 31 3 297 301 10.1016/j.reprotox.2010.07.012 20850519 PMC3025269

[B22] Lukaszewicz-Hussain A [Paraoxonase activity and lipid peroxides concentration in serum of rats subchronically intoxicated with chlorpyrifos--organophosphate insecticide] Med Pr 2012 63 5 559 564 23373324

[B23] Shobha TR Prakash O Glycosuria in organophosphate and carbamate poisoning J Assoc Physicians India 2000 48 12 1197 1199 11280228

[B24] Zadik Z Blachar Y Barak Y Levin S Organophosphate poisoning presenting as diabetic ketoacidosis J Toxicol Clin Toxicol 1983 20 4 381 385 10.3109/15563658308990606 6655779

[B25] Akyildiz BN Kondolot M Kurtoglu S Akin L Organophosphate intoxication presenting as diabetic keto-acidosis Ann Trop Paediatr 2009 29 2 155 158 10.1179/146532809X440789 19460270

[B26] Kumar KJ Nayak N Organophosphorus poisoning presenting as diabetic ketoacidosis Indian Pediatr 2011 48 1 74 10.1007/s13312-011-0001-5 21317476

[B27] Karami-Mohajeri S Abdollahi M Toxic influence of organophosphate, carbamate, and organochlorine pesticides on cellular metabolism of lipids, proteins, and carbohydrates: a systematic review Hum Exp Toxicol 2010 30 9 1119 1140 21071550 10.1177/0960327110388959

[B28] Hasler RM Nuesch E Juni P Bouamra O Exadaktylos AK Lecky F Systolic blood pressure below 110mmHg is associated with increased mortality in penetrating major trauma patients: multicentre cohort study Resuscitation 2012 83 4 476 481 10.1016/j.resuscitation.2011.10.018 22056618

[B29] Gaspari RJ Paydarfar D Respiratory failure induced by acute organophosphate poisoning in rats: effects of vagotomy Neurotoxicology 2009 30 2 298 304 10.1016/j.neuro.2009.01.006 19428503

[B30] Chuang FR Jang SW Lin JL Chern MS Chen JB Hsu KT QTc prolongation indicates a poor prognosis in patients with organophosphate poisoning Am J Emerg Med 1996 14 5 451 453 10.1016/S0735-6757(96)90148-5 8765106

